# The effect of the stress autism mate app on perceived stress, coping, and resilience in adults with autism: a single-case experimental design

**DOI:** 10.3389/fpsyt.2024.1347947

**Published:** 2024-06-13

**Authors:** Kirsten Hoeberichts, Yvette Roke, Irene Niks, Peter N. van Harten

**Affiliations:** ^1^ Expertise Centre Specialised in Autism Spectrum Disorder, GGz Centraal, Almere, Netherlands; ^2^ Department of Psychiatry and Neuropsychology, School of Mental Health and Neuroscience, Maastricht University, Maastricht, Netherlands; ^3^ Department Work, Health & Technology, The Netherlands Organization for Applied Scientific Research (TNO), Leiden, Netherlands; ^4^ Department of Psychiatry, GGz Centraal, Amersfoort, Netherlands

**Keywords:** autism, perceived stress, coping, resilience, stigma, quality of life, mental health, digital intervention

## Abstract

**Introduction:**

The mobile health application “Stress Autism Mate” (SAM) was designed to support adults with autism in identifying and managing daily stress. SAM measures stress four times daily, provides a daily and weekly stress overview, and provides personalised stress reduction advice. This study aimed to assess the effectiveness of SAM over four weeks in reducing perceived stress and internalised stigma, and enhancing coping self-efficacy, quality of life, and resilience among adults with autism.

**Methods:**

Using an A1-B-A2 single-case experimental design, the effect of using SAM on adults with autism was assessed. The phases consisted of A1; treatment as usual (TAU), B; introducing SAM, and finally A2; follow-up with TAU and without the use of SAM. Each phase lasted four weeks, and data were collected via questionnaires before and after each phase. Linear mixed models were used for data analysis.

**Results:**

Results show significant reductions in perceived stress levels, increased coping self-efficacy, and improved perceived health and psychological well-being after using SAM. Furthermore, increased resilience, and decreased internalised stigma were reported after follow-up.

**Discussion:**

In conclusion, this study highlights SAM as a valuable tool for empowering adults with autism to reduce stress and internalised stigmaand to improve coping self-efficacy, psychological well-being, and resilience.

## 1 Introduction

Individuals with autism generally experience high levels of stress (1–3). This may be due to certain characteristics of autism, such as persistent deficits in social communication and interaction, and restricted, repetitive patterns of behaviour, interests, or activities (4). In addition, life events, maladaptive coping strategies, and differences in sensory processing are other potential factors contributing to high levels of perceived stress in adults with autism (1, 5).

Perceived stress refers to an individuals’ feelings and thoughts related to the stressfulness of their daily life and their ability to overcome these stressful events (6, 7). Experiencing stress in daily life serves an important function as moderate stress levels can motivate and prepare an individual to respond effectively to challenges and demands. However, when the level of stress exceeds an individuals’ capacity to cope effectively, it can remain elevated and develop into chronic stress. Chronic stress can have various adverse consequences on both physical and psychological well-being, such as elevated blood pressure, palpitations, increased risk of depression, and anxiety (8, 9). Furthermore, high levels of perceived stress are associated with lower resilience and reduced quality of life (3, 10). Resilience is “a personality characteristic that moderates the negative effects of stress and promotes positive adaption” (11), while quality of life refers to individuals’ perceptions of their position in life to their goals, expectations, standards, and concerns (12). In sum, actively reducing perceived stress levels and preventing chronic stress is important for improving mental and physical well-being, particularly for individuals with autism.

Coping, defined as behavioural or cognitive efforts to manage stressful situations (13), plays a crucial role in stress reduction. Effective, positive coping strategies not only enable individuals to manage and reduce stress but also enhance resilience. Increased resilience, in turn, improves the ability to apply adaptive coping strategies in stressful situations (10, 14–16). In addition, Holubova et al, (17) argues that positive coping strategies can decrease internalised stigma. Internalised stigma is the harmful psychological impact that occurs when a person absorbs negative prejudice and discrimination about people with mental illness and starts to believe and apply them to themselves (18, 19). Higher levels of internalised stigma are associated with lower levels of quality of life, hope, self-esteem, and social support (20, 21).

Early recognition of stress so that effective coping strategies can be used to reduce stress is necessary to prevent stress from becoming chronic. An important issue, however, is that individuals with autism may find it quite challenging to identify and effectively cope with stress (1, 3). As such, individuals with autism may benefit from a tool that helps them identify stress so that they can manage it.

In a previous pilot study (22), a mobile application called ‘Stress Autism Mate’ (SAM) aimed at supporting adults with autism in identifying and effectively managing their stress levels was developed and tested. SAM uses questionnaires four times a day to assess activities and stress levels and provides personalised stress reduction advice and visual feedback charts based on research-based algorithms. The current study aimed to evaluate the effectiveness of SAM in reducing perceived stress and internalised stigma, improving coping self-efficacy and quality of life, and fostering resilience in adults with autism, specifically adults who were not involved in the development of the app.

## 2 Materials and methods

### 2.1 Study design

**Figure 1:**
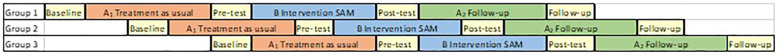
Visualisation of the study design. A1: control phase with TAU; B: intervention phase with use of SAM; A2: follow-up with TAU.

An A_1_-B-A_2_ single-case experimental design (SCED) (23) was used to examine the effects of the intervention without the use of a control group (**Figure 1**). Participants received treatment as usual (TAU) in phase A_1_, B represents the introduction of the intervention SAM, and A_2_ represents a follow-up period with TAU, without the use SAM. Each phase lasted four weeks and began and ended with a questionnaire. The use of a SCED allowed the study to focus on monitoring individual changes in outcome measures over time, with participants acting as their own controls. This design is considered highly appropriate for assessing behavioural change in ‘real world’ settings (24, 25). To minimise the risk of history bias, where external factors unrelated to the intervention may influence the outcome measure, participants were randomised into three groups by a random numbers generator, with each group starting the trial two weeks apart, as shown in **Figure 1**. The randomisation was done by using Microsoft Excel. This design was chosen to increase the validity of the study results and to ensure that observed effects are more likely to be due to the intervention itself.

### 2.2 Participants

The study included participants based on specific inclusion criteria: (a) a diagnosis of autism according to the DSM-5 (4) and the diagnostic guideline of the Dutch Association of Psychiatry (NVVP); (b) age = 18 years. Exclusion criteria were: (a) having an IQ lower than 85 according to the Wechsler Adult Intelligence Scale IV Dutch (WAIS-IV-NL); (b) previous involvement in research or familiarity with SAM (22); (c) inability to understand the Dutch language; and (d) inability to use a smartphone. All 36 participants in the study were being treated at Emerhese, a centre of expertise for autism in the Netherlands. During the study, two participants dropped out while using SAM and reported feeling more stressed when using SAM; it is not known what aspect of SAM or participation in the study was so stressful that they dropped out.

The participant group consisted of eighteen females and sixteen males with a mean age of respectively 36 years (SD 13.2) and 42 years (SD11.6). Specific data on socioeconomic status and race/ethnicity were not recorded.

### 2.3 Intervention

**Figure 2:**
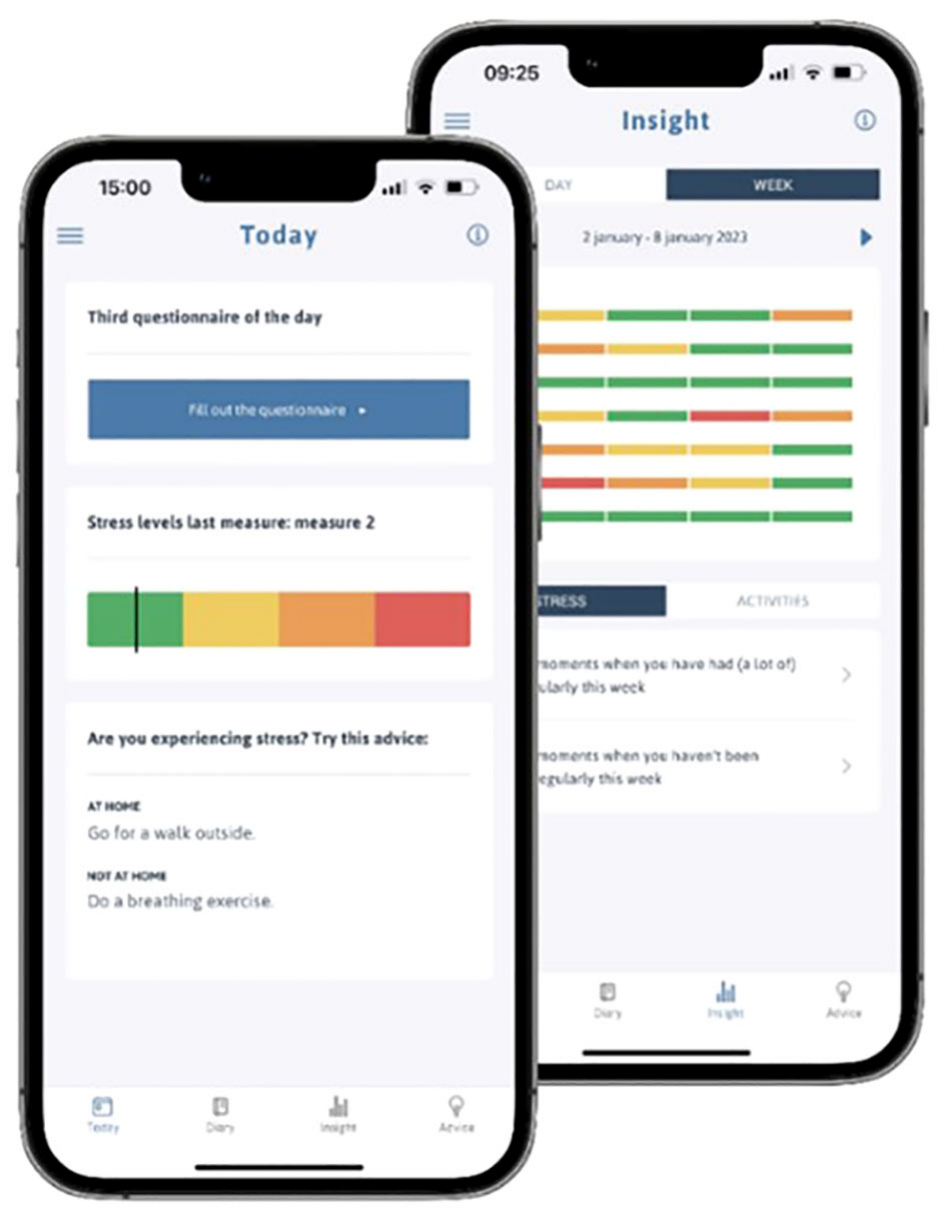
Screenshots of the homepage and the visual overview page of SAM.

SAM is a personal self-help mobile application designed to help users identify and manage everyday stress. SAM is developed through a collaborative process with its end-users, ensuring that their needs and perspectives are integrated into the design and functionality of the app. SAM sets a questionnaire four times a day that includes questions about the last four hours’ activities and stress-related questions. The research-based algorithm in the app calculates a stress level based on the user’s answers, which is then verified by the user’s subjective experience. The app provides personalised coping advice to reduce the measured stress level. In addition, SAM generates a visual feedback graph, shown in **Figure 2**, which displays daily and weekly stress levels. SAM is freely available in app stores across Europe. More detailed information on the features and development of SAM can be found in the previous publication by Hoeberichts et al. (22), and at https://www.stressautismmate.nl/.

### 2.4 Questionnaires

The effectiveness of SAM is evaluated by the following outcome measures; (i) perceived stress, (ii) coping self-efficacy, (iii) quality of life, (iv) resilience and (v) internalised stigma. These variables are assessed using valid and reliable self-report questionnaires administered at the specified measurement points. The internal consistency of the measures was assessed using McDonald’s omega coefficient.

#### 2.4.1 Perceived stress; McDonald’s ω = .822

Perceived stress was assessed using the Perceived Stress Scale (PSS) (26, 27). The PSS has been validated in adults with autism (1), and has shown good internal consistency. The questionnaire consists of 10 items, rated on a five-point Likert scale ranging from “Never” to “Very often”. Four items were reverse scored. The sum of the 10 items results in a total score, with higher scores indicating higher levels of perceived stress.

#### 2.4.2 Coping self-efficacy; McDonald’s ω = .884

Coping self-efficacy was assessed using the Coping Self-Efficacy Scale (CSES) (28). The CSES has three subscales, respectively problem-focused coping, stopping unpleasant emotions and thoughts, and getting support from friends and family. Participants rated their responses on a scale ranging from 0 to 10, i.e., from “I am not able at all” to “I am very able”. Sum scores of the subscales were calculated, with higher scores indicating greater coping self-efficacy.

#### 2.4.3 Quality of life*;* McDonald’s ω = .898

Quality of life was assessed using the World Health Organisation Quality of Life Questionnaire (WHOQoL-BREF) (12). In addition to the separate questions “*How would you rate your quality of life?*” and “*How satisfied are you with your health*?”, three domains were used: physical health, psychological well-being, and social relationships. All items were scored on a five-point Likert scale ranging from “Very poor” to “Very good”. For three items, the scores were reversed. Mean scores were calculated for each domain, with higher mean scores indicating a better score for the (sub)scale topic.

#### 2.4.4 Resilience; McDonald’s ω = .872

Resilience was measured using the Resilience Scale (RS-25) (11, 29, 30). This widely used questionnaire consists of two subscales: Personal Competence (PC) and Acceptance of Self and Life (ASL). Items were scored on a four-point Likert scale ranging from “Strongly disagree” to “Strongly agree”. The results of this questionnaire are a total score, and two subscale totals, with higher scores indicating higher levels of resilience.

#### 2.4.5 Internalised stigma*;* McDonald’s ω = .771

To address internalised stigma, the Internalised Stigma Mental Illness scale (ISMI-10) (31) was used. The questionnaire consists of 10 items, each rated on a four-point Likert scale ranging from “Strongly disagree” to “Strongly agree”. For two items, the scores were reversed. The mean score was calculated based on the 10 items, with a higher mean score indicating a higher level of internalised stigma.

### 2.5 Procedure

Participants were informed about the study through their therapist and a patient information letter. Potential participants who met the inclusion criteria were contacted by the research group for a face-to-face or online appointment to provide further information about the study and to obtain written informed consent. The study consisted of three phases, which are described in detail below.

#### 2.5.1 Control phase: A1

At baseline, each participant attended an appointment with the researcher to complete the first questionnaire as a baseline measurement. This was followed by a four-week control phase during which participants continued their usual treatment with no additional study requirements. At the end of the control phase, participants attended a second appointment to complete the second questionnaire (pre-test).

#### 2.5.2 Intervention phase: B

During the second appointment, the participants installed SAM on their mobile phones together with the researcher. The researcher explained the settings and functionalities of the app in detail. During the four-week intervention phase, participants used SAM four times a day, seven days a week, while continuing their usual treatment. A helpdesk was available to provide technical support for the app. At the end of the intervention phase, participants attended a third appointment to complete the third questionnaire (post-test). They also deleted SAM from their mobile phones and had the opportunity to give feedback on the app.

#### 2.5.3 Follow-up phase: A2

After completing the third questionnaire, participants entered the follow-up phase, which lasted another four weeks. During this phase, participants continued with their usual treatment and were not allowed to use SAM. The trial ended with a fourth and final appointment, where participants completed the fourth questionnaire (follow-up). They were thanked for their participation in the study and could continue to use SAM for personal use if they wished.

### 2.6 Data analyses

Data analyses were performed using linear mixed effect models (LMM) in IBM SPSS version 28. LMM was chosen because it can account for within-subject variance over time, making it suitable for analysing the data from the SCED design used in this study (32, 33). All data were pseudonymised, and only the researchers had access to the identification key. Data from the two participants who dropped out were excluded from the analyses.

Prior to the analysis of the outcome measures, two fundamental aspects were examined. Firstly, LMM group analyses were conducted between groups to determine whether there was a significant difference between the groups for each parameter. If such an effect was found, there is a possibility of a history bias, which means that external factors unrelated to the intervention might influence the outcome measure. Secondly, we examined whether the control phase assumption was met for each outcome measure. This assumption implies that if there is no significant variation between baseline and pre-test measurement, it can be assumed that no spontaneous change over time occurs during TAU on the outcome measure in question. This analysis was essential to ensure that any changes in outcome measures after using the intervention SAM could be attributed to the intervention. Should a significant change be observed during the control phase, that parameter was excluded from further analysis, as it would then be challenging to determine whether any potential effect could be attributed to the use of the SAM app or to spontaneous changes over time and TAU. If the condition was met, and no significant change was found during the control phase, baseline was chosen as the reference point against which post-test and follow-up measurements were compared.

In each model, the measure was treated as a fixed effect, allowing for differentiation for each participant. A significance level of p < 0.05 was used for all statistical tests. For each significant outcome, Cohen’s d effect sizes were calculated based on the mean change scores from baseline to post-test and follow-up.

## 3 Results

A total of 34 participants successfully completed all questionnaires at baseline, pre-test, post-test, and follow-up. The results of the study are summarised in **Tables 1**, **2**.

**Table 1:** The means and standard deviations of the PSS, CSES, WHOQOL-bref, RS-25 and ISMI-10 at baseline, pre- and post-test and follow-up.

		Baseline	Pre-test	Post-test	Follow-up
Outcome variable	Range	Mean (SD)	Mean (SD)	Mean (SD)	Mean (SD)
Perceived stress					
**PSS**	0 – 50	22.56 (5.40)	21.79 (5.40)	19.18 (5.03)	19.76 (6.58)
Coping self-efficacy					
**Total CSES**	0 – 130	53.62 (17.63)	54.03 (15.97)	61.38 (20.73)	60.59 (21.95)
**Problem focused coping**	0 – 50	25.59 (10.12)	25.97 (8.50)	29.79 (11.16)	28.41 (11.07)
**Manage emotions**	0 – 40	15.76 (6.39)	14.82 (6.56)	17.44 (7.14)	17.94 (7.85)
**Social support**	0 – 20	12.26 (6.06)	13.24 (5.12)	14.15 (5.45)	14.24 (6.25)
WHOQOL					
**Estimated quality of life**	0 – 5	2.94 (.98)	3.26 (.99)	–	–
**Perceived health**	0 – 5	2.79 (1.01)	3.00 (1.07)	3.12 (.91)	3.26 (1.08)
**Physical well-being**	0 – 5	3.16 (.79)	3.21 (.78)	3.28 (.71)	3.34 (.70)
**Psychological well-being**	0 – 5	2.81 (.73)	2.88 (.76)	3.04 (.73)	3.06 (.73)
**Social relationships**	0 – 5	3.23 (.83)	3.24 (.82)	3.31 (.86)	3.37 (.70)
Resilience					
**Total RS**	1 – 125	78.12 (12.70)	79.88 (13.19)	80.88 (12.69)	82.38 (14.42)
**Personal Competence**	1 – 85	55.24 (8.14)	56.09 (8.72)	56.68 (8.98)	57.62 (10.25)
**Acceptance of self and life**	1 – 40	22.88 (5.51)	23.79 (5.58)	24.21 (4.93)	24.76 (5.48)
Internalised stigma					
**ISMI**	1 – 4	2.08 (.51)	2.04 (.49)	2.04 (.36)	1.97 (.40)

PSS, Perceived Stress Scale; CSES, Coping Self-Efficacy Scale; WHOQOL-bref, World Health Organization Quality of Life; RS, Resilience Scale; ISMI, Internalised Stigma of Mental Illness Inventory.

**Table 2:** The results of the linear mixed model analyses for the PSS, CSES, WHOQOL-bref, RS-25 and ISMI-10 at baseline to pre-test, baseline to post-test and baseline to follow-up, with signicant results in bold.

	Baseline to pre-test		Baseline to post-test		Baseline to follow-up	
**Outcome variable**	β	*SE*	β	*SE*	β	*SE*
Perceived stress						
**PSS**	-.76	.59	-3.38**	.81	-2.79*	1.17
Coping self-efficacy						
**Total CSES**	.41	2.33	7.76**	2.51	6.97**	2.48
**Problem focused coping**	.38	1.32	4.21**	1.38	2.82	1.25
**Manage emotions**	-.94	1.05	1.68	1.13	2.18	1.10
**Social support**	.97	.69	1.88*	.74	1.97**	.71
WHOQOL						
**Estimated quality of life**	.32*	.12	–	–	–	–
**Health perception**	.21	.61	.32*	.15	.47**	.16
**Physical health**	.04	.08	.11	.10	.18	.09
**Psychological well-being**	.06	.07	.23**	.07	.25**	.08
**Social relationships**	.00	.12	.08	.10	.14	.11
Resilience						
**Total resilience**	1.76	1.13	2.76	1.60	4.26*	1.58
**Personal Competence**	.85	.77	1.44	1.16	2.38*	1.16
**Acceptance of self and life**	.91	.51	1.32*	.63	1.88**	.64
Internalised stigma						
**Mean ISMI**	-.03	.05	-.05	.07	-.11*	.05

PSS, Perceived Stress Scale; CSES, Coping Self-Efficacy Scale; WHOQOL, World Health Organization Quality of Life; ISMI, Internalised Stigma of Mental Illness Inventory; β, Estimate; SE, standard error; *p < 0.05; **p < 0.01 significant higher or lower scores compared to baseline.

### 3.1 Basic assumptions

#### 3.1.1 Group analysis

The data were analysed at baseline to assess whether the basic assumptions were met, which included no significant differences between the groups. However, significant differences were found in group two compared to groups one and three for problem-focused coping self-efficacy (β = -6.87, *SE* = 2.84, p = .022 and β = 9.81, *SE* = 2.98, p = .002) and perceived health as measured by the WHOQoL-BREF (β = -.81, *SE* = .32, p = .016 and β = .86, *SE* = .34, p = .015). Additionally, differences were observed in group one compared to groups two and three for internalised stigma (β = .33, *SE* = .13, p = .019 and β = .31, *SE* = .14, p = .036). No significant effects were found for the other (sub)scales.

#### 3.1.2 Control phase

The data were analysed to assess whether the basic assumptions were met, which included no significant difference between the (sub)scale scores at baseline and pre-test. Most of the (sub)scales met these conditions, except for the WHOQoL-BREF ‘estimated quality of life’ question, which improved significantly between pre-test and baseline (beta = .32, *SE* = .12, p = .014). As this question did not meet the baseline assumptions, it was not analysed further.

### 3.2 Effects of the intervention

#### 3.2.1 Perceived stress

Participants’ perceived stress decreased significantly at post-test compared to baseline (β = -3.38, *SE* = 0.81) and follow-up (β = -2.79, *SE* = 1.17), with medium (*d* = .65) and small (*d* = .47) effect sizes, respectively.

#### 3.2.2 Coping self-efficacy

Coping self-efficacy improved significantly compared to baseline at post-test (β = 7.76, *SE* = 2.51) and at follow-up (β = 6.97, *SE* = 2.48). These effect sizes are both small (*d* = .40 and *d* = .35). The ability to ask friends and family for support improved significantly compared to baseline at post-test (β = 1.88, *SE* = .74) and follow-up (β = 1.97, *SE* = .71). The effect sizes at post-test and follow-up are both small (*d* = .33 and *d* = .32 respectively). Problem-focused coping improved significantly compared to baseline at post-test (β = 4.21, *SE* = 1.38) with a small effect size (*d* = 0.40). This effect diminished at follow-up. The ability to stop unpleasant emotions and thoughts did not have a significant effect compared to baseline at post-test and follow-up.

#### 3.2.3 Quality of life

Perceived health improved significantly compared to baseline at post-test (β = .32, *SE* = .15) and follow-up (β = .47, *SE* = .16), both with small effect sizes (*d* = .34 and *d* = .45 respectively). Psychological well-being improved significantly compared to baseline at post-test (β = .23, *SE* = .07) and at follow-up (β = .25, *SE* = .08), both with small effect sizes (respectively *d* = .31 and *d* = .34). Physical health and social relationships showed no significant effects at either post-test or follow-up compared to baseline.

#### 3.2.4 Resilience

Resilience improved significantly at follow-up compared to baseline (β = 4.26, *SE* = 1.58), with a small effect size (*d* = 0.31). There was no significant effect at post-test. Personal competence at follow-up (β = 2.38, *SE* = 1.16) improved significantly compared to baseline, with a small effect size (*d* = .26). Acceptance of self and life improved significantly compared to baseline at post-test (β = 1.32, *SE* = .63) and at follow-up (β = 1.88, *SE* = .64). These effect sizes are both small (*d* = 0.25 and *d* = 0.34 respectively).

#### 3.2.5 Internalised stigma

Internalised stigma decreased significantly at follow-up compared to baseline (β = -.11, *SE* = .05), with a small effect size (*d* = .23). There was no significant effect seen at post-test.

## 4 Discussion

This study demonstrates the effectiveness of SAM as a tool to support adults with autism in identifying and managing daily stress. Surprisingly, this relatively easy to implement and straightforward intervention not only affected perceived stress, but also had a positive effect on all other outcome variables. That is, after using SAM for four weeks, participants experienced a significant decrease in perceived stress, improved coping self-efficacy, enhanced perceived health, and better psychological well-being after both the intervention and follow-up phase as well as increased resilience and a decrease in internalised stigma at follow-up. However, as discussed in the limitations section, it’s important to acknowledge that the latter findings may be susceptible to historical bias.

The results were broadly consistent with our previous pilot study (22), with SAM showing an immediate and sustained effect on most outcome measures. At first, participants reported a reduction in perceived stress, suggesting that SAM can cause a positive shift in negative thoughts and feelings associated with stressful events (6, 7). In addition, the intervention led to noteworthy improvements in coping self-efficacy, particularly in seeking social support to cope with challenges. This aspect is particularly relevant given that individuals with autism often face difficulties in social communication (2). The observed enhancements in perceived stress and coping self-efficacy may be attributed to SAM’s functional design. SAM operates by prompting users to pause and assess their well-being and stress levels throughout the day and offers personalised advice on how to manage stress. Additionally, the app provides insight into individual stress patterns across time and activities. By raising awareness of stress patterns, encouraging support-seeking behaviours, and reducing overall stress levels, SAM appears to empower individuals to actively seek and benefit from social support.

In addition, participants reported improvements in their perceived health and psychological well-being. In the context of quality of life, psychological well-being includes negative and positive emotions, self-worth, and self-esteem (12). The literature suggests interrelationships among self-esteem, coping, and perceived stress. Namely, reduced stress levels may lead to improved self-esteem, while higher levels of self-esteem may act as a buffer against the impact of stressful events and promote the use of adaptive coping strategies (34, 35). Consistent with the literature, this study shows that SAM supports users in reducing perceived stress, thereby improving coping self-efficacy, and potentially boosting self-esteem. Furthermore, loneliness serves as a significant negative indicator of psychological well-being (36). As coping self-efficacy for seeking social support improves after using SAM, feelings of loneliness may decrease, leading to an overall improvement in psychological well-being.

Two outcome measures showed a significant effect only following the four-week follow-up. First, there was an improvement in resilience. The literature indicates a reduction in perceived stress and the use of effective coping strategies are known to improve resilience (10, 14). As such, the observed positive effect of SAM on resilience may be due to the initial reduction in perceived stress and improvement in coping self-efficacy, both of which are facilitated using SAM. It is possible that these factors contribute to a gradual increase in resilience over time, resulting in the significant improvement in resilience being solely measurable at follow-up. Secondly, a reduction in internalised stigma was documented. Previous research has found a negative relationship between self-esteem and internalised stigma (20, 21, 37). As previously discussed above, the results indicate that the use of SAM may lead to an increase in psychological well-being, which includes self-esteem. This increased self-esteem may subsequently contribute to a reduction in internalised stigma. Just like resilience, it is possible that the secondary effect of SAM may take longer to manifest, which may explain why the effect of decreased internalised stigma was only observed at the follow-up assessment. However, it is important to note that these explanations are somewhat speculative and that it is also possible that both changes are spontaneous and not necessarily attributable to the use of the SAM app.

One variable that only showed an effect immediately after SAM use and not at follow-up was problem-focused coping self-efficacy, which can be defined as ‘effectively managing challenging aspects of stressful situations and using strategies to reduce the perceived severity of the problem’ (28). This suggests that active use of SAM may have an immediate positive effect on problem-focused coping self-efficacy, but this effect may diminish over time if individuals stop using SAM, highlighting the importance of active use of SAM as an external tool for problem-focused coping in particular.

Unfortunately, SAM did not have a significant effect on self-efficacy for managing emotion and perceived physical health and social relationships, neither immediately after SAM use nor at follow-up. This may be because the stress reduction advice provided by SAM focuses primarily on active stress reduction, such as finding distraction, doing relaxation exercises, or seeking social support, rather than specifically targeting emotion regulation techniques, or improving physical health. In addition, SAM primarily reduces perceived stress, which may be a relatively minor contributor to perceptions of physical health and social relationships. Finally, it is also important to consider that the duration of the intervention and the follow-up period may not have been long enough to fully observe the effects of SAM on emotional coping self-efficacy, perceived physical health and social relationships.

Finally, it is worth noting that the two participants who dropped out did so while using SAM and reported feeling more stressed when using SAM. Unfortunately, the specific underlying factors responsible for this increased stress, whether due to particular aspects of SAM or participation in the study itself, remain unidentified. This highlights the importance of recognising that not everyone may derive the same benefits from an application such as SAM, and that the potential risk of increased stress when using it should be carefully considered.

### 4.1 Strengths and limitations

This study is of significant practical importance as it provides scientific evidence of the highly beneficial effects of SAM on its users. This research is particularly relevant in the context of the wider conversation about mental health support for people with autism, as it addresses a critical need for evidence-based, personalised stress management interventions that take into account the unique challenges faced by this population. SAM’s multilingual capability and Europe-wide availability add to its importance as a widely applicable tool.

Another strength of this study is the use of a SCED design. SCED allows all participants to experience the potential real-life benefits of the intervention (38). By eliminating the need for a comparison group, this design minimises variation between groups and facilitates the assessment of treatment response at an individual level (39). A third strength is the relatively low dropout rate of 5.56% observed in this study.

Nevertheless, there are several limitations that should be taken into account when interpreting the findings. Firstly, the single-centre nature of the study may have resulted in a homogeneous group in terms of treatment and stress management. However, it is important to note that although the participants came from a single centre, the intervention took place in their own everyday situations outside the treatment setting. This real-world context may have introduced a variety of factors that could have influenced the results, making it less likely that the group remained completely homogeneous. It should also be considered that the participants in this study were already being treated at GGz Centraal, a tertiary treatment centre for autism. This means that they had already received some form of treatment and stress management prior to the study. Despite their previous treatment experience, the use of SAM still yielded significant effects. This suggests that SAM can be beneficial even for individuals who have already received treatment before. Therefore, while the single-centre nature of the study may have initially raised concerns about homogeneity, the real-world context of the intervention and the inclusion of participants with a treatment background contribute to the robustness and relevance of the findings.

Secondly, to minimise the risk of history bias, where external factors unrelated to the intervention may influence the outcome measure, participants were randomly assigned to three groups. Each group started the trial two weeks apart. In summary, significant differences were observed between the groups for the (sub)variables of problem-focused coping self-efficacy, perceived health, and internalised stigma. This highlights the need to carefully interpret the results of these variables. That is, external factors, such as illness or financial stressors, unrelated to the intervention may have influenced the observed outcomes of problem-focused coping self-efficacy, perceived health, and internalised stigma. Regarding the other outcome measures, no differences were observed between the groups, which lends support to the hypothesis that the observed effects were due to SAM use.

Finally, it is important to consider the potential response bias that could arise from the use of self-report questionnaires in all four measures, as noted by Moulds et al, (40). This bias, commonly found in self-report measures, could lead to participants providing less than truthful responses. Various steps were taken to minimise this bias. Firstly, response fatigue was avoided as the time taken to complete the questionnaire, this was limited to a maximum of 45 minutes. Moreover, the use of simple and accessible language in the questionnaires aimed to ensure clarity and ease of understanding for participants, thereby reducing the probability of biased responses.

### 4.2 Implications and future directions

The results of both the current study and the previous study (22) provide promising evidence for the effectiveness of a stress management app such as SAM for individuals with autism. Furthermore, SAM has received consistently positive feedback from its users suggesting that SAM is also perceived as effective by its users. This highlights the potential benefits of SAM in reducing perceived stress and increasing coping self-efficacy in adults with autism. Overall, these positive findings suggest that the use of SAM may also be beneficial in the treatment of adults with autism. Moreover, by encouraging SAM users to discuss their daily and weekly stress levels with their therapist, therapists can gain valuable insight into an individual’s stress patterns, enabling them to tailor interventions specifically designed to improve the daily lives of people with autism and make their lives more manageable.

Future research could benefit from further exploration of the potential benefits of SAM for other target groups, which could open up new avenues for research and application, such as younger people with autism, people from different cultural backgrounds or individuals with other mental health conditions, such as anxiety or borderline personality disorder. Replicating the study in a diverse population would help determine whether the observed results hold true in different cultural and situational contexts. Furthermore, the influence of the duration of the intervention on the effectiveness of SAM should also be considered. The study used a four-week intervention period. However, it is possible that beneficial effects of SAM use could be seen within alternative time frames. For instance, a shorter intervention period, such as two weeks, or a longer period, such as six weeks, might also produce positive effects that are worth exploring. A trial exploring this could help determine the optimal length of time to use SAM to achieve the desired effects. Finally, another avenue for future research is a study that includes assessments at multiple time points after the intervention, such as three months, six months and one year, which would provide valuable insights into whether the positive outcomes of SAM are sustained over time. This longitudinal approach would contribute to a full understanding of the lasting effects and practical implications of SAM in real-world settings.

In conclusion, this study shows that the relatively easy to implement intervention SAM seems remarkably effective in reducing perceived stress and increasing coping self-efficacy in adults with autism. Results also indicate that by effectively managing stress, individuals with autism experience improved psychological well-being, resilience and decreased internalised stigma. Therefore, we believe that SAM shows promise as a valuable tool to support individuals with autism in identifying, managing, and reducing stress in their daily lives.

## Data availability statement

The raw data supporting the conclusions of this article will be made available by the authors, without undue reservation.

## Ethics statement

The studies involving humans were approved by Medical ethics review committee Isala Zwolle. The studies were conducted in accordance with the local legislation and institutional requirements. The participants provided their written informed consent to participate in this study.

## Author contributions

KH: Methodology, Investigation, Writing – original draft. YR: Investigation, Writing – review & editing. IN: Writing – review & editing. PH: Supervision, Writing – review & editing.
